# Clinical problems of patients with cachexia due to chronic illness: a congress report

**DOI:** 10.1002/ehf2.13052

**Published:** 2020-10-04

**Authors:** Sara Hadzibegovic, Philipp Sikorski, Sophia K. Potthoff, Jochen Springer, Alessia Lena, Markus S. Anker

**Affiliations:** ^1^ Division of Cardiology and Metabolism, Department of Cardiology Charité ‐ Campus Virchow Klinikum (CVK) Berlin Germany; ^2^ Department of Cardiology Campus Benjamin Franklin (CBF), Charité University Medicine Berlin Germany; ^3^ Berlin Institute of Health Center for Regenerative Therapies (BCRT) Berlin Germany; ^4^ DZHK (German Centre for Cardiovascular Research) Berlin Germany

## Introduction

Cachexia is a complex and multifactorial comorbidity characterized by a metabolic imbalance.^1^, ^2^ It is defined as a body weight loss ≥5% in the last 12 months in patients with a chronic illness who also suffer from at least three of the following five symptoms: fatigue, decreased muscle strength, low fat‐free mass index, anorexia, and/or altered biochemistry (haemoglobin < 12 g/dL, serum albumin < 3.2 g/dL, increased interleukin 6, or C‐reactive protein).^3^ It is influenced by immunological,^4^ neurohormonal,^5^ and mucointestinal^6^ impairments and aggravated by factors such as malabsorption and dietary deficiencies.^7^, ^8^ Cachexia is frequently seen in different chronic diseases^9^: in 50–80% of advanced cancer patients,^10^ 30–50% of dialysis patients,^11^ 20–30% of chronic obstructive pulmonary disease patients,^12^ 20–30% of rheumatoid arthritis patients,^13^ 10% of patients after neurologic stroke,^14^ and 10–20% of chronic heart failure (CHF) patients.^15^, ^16^ As an example, CHF patients share many risk factors with cachexia that also contribute to its development: high prevalence of comorbidities,^17^, ^18^ immunoflammatory alterations,^19^ worsening nutritional status,^20^ and a sedentary lifestyle with a high risk for frailty^21^ due to a decrease of daily life activities^22^ or frequent rehospitalizations.^23^ Hence, wasting disorders are associated with poorer quality of life,^24^ longer hospitalization rates,^25^ and increased mortality.^26^, ^27^, ^28^

Therefore, there is a need for annually meetings, in person or digital, where clinicians and basic researches can share their knowledge on the topic. Over 400 participants from more than 35 countries attended the ‘12^th^ International Conference on Cachexia, Sarcopenia and Muscle Wasting’, which was held in Berlin, Germany, from 6 to 8 December 2019. It provided a great platform for new updates about cachexia and muscle wasting disorders. Among a variety of lectures and poster presentations, one special clinical session was dedicated to the most frequent symptoms in patients with cachexia and their treatments: swallowing problems, pain, depression, muscle weakness, fatigue, and shortness of breath.

## Swallowing problems

In the first lecture of the session, Professor Hidetaka Wakabayashi from Yokohama, Japan, thematized swallowing problems in cachectic patients with cancer. Strongly depending on the cancer entity and stage, they occur in 30–90% of cancer patients during anticancer treatment.^29^ Head and neck cancer, prior radiotherapy, nasogastric tubes, and low skeletal mass are the most frequent contributing factors to swallowing problems.^30^ Among hospitalized patients, those with cardiovascular comorbidities like arterial hypertension, acute myocardial infarction, and CHF have a high risk to develop oropharyngeal dysphagia.^31^ Professor Wakabayashi highlighted the importance of diagnosing sarcopenic dysphagia. It is characterized by four major criteria: whole body sarcopenia, dysphagia, reduced swallowing muscle mass on imaging tests, and an exclusion of all other causes for dysphagia.^32^ This can be found in up to 30% of patients in dysphagia rehabilitation.^33^ Therefore, especially in older patients with sarcopenia and dysphagia, hand grip strength, gait speed, and swallowing function should be routinely tested in order to identify patients with sarcopenic dysphagia. According to the speaker, the treatment of sarcopenic dysphagia includes three major components: exercise,^34^ nutritional,^35^ and dysphagia^36^ rehabilitation. An early rehabilitation with nutritional support consisting of approximately 35 kcal/kg/day is important for prevention and treatment. It also involves frequent monitoring of the body function, quality of life, and an assessment of frailty in order to obtain early diagnosis and valid interventions.^37^ A dysphagia rehabilitation^36^ consists of oral hygiene with dental hygienist intervention,^38^ resistance exercise,^39^ and food modification according to the International Dysphagia Diet Standardization Initiative framework.^40^ At last, the speaker pointed out that sarcopenia can also have an iatrogenic cause,^41^ if for example the daily energy intake from hospital meals is too low during dysphagia rehabilitation.^33^ Therefore, early rehabilitation, early oral intake, and appropriate nutritional support are important to prevent sarcopenia and dysphagia.

## Pain

In the next lecture, Associate Professor Florian Strasser from St Gallen, Switzerland described the different kinds of pain syndromes, which frequently occur in cachectic patients with advanced cancer. Pain can be caused by cancer disease itself^42^ or appear due to anticancer treatment, for example, chemotherapy induced neuropathy.^43^, ^44^ It also frequently occurs in chronic diseases like low back pain syndromes,^45^ multiple sclerosis,^46^ rheumatic diseases,^47^ chronic obstructive pulmonary disease,^48^ or transits post surgically from acute to chronic pain.^49^ In a descriptive cross‐sectional study with 62 CHF patients with NYHA class II‐IV, pain was frequently observed. ^50^ Ninety‐eight per cent of the patients described unspecified pain in the past month, and 66% had pain in the last 24 h. The character of pain in 1886 CHF patients was assessed in another interventional study. Eighty per cent of patients reported at least some chest pain, and the authors concluded that it was unlikely only due to angina pectoris.^51^ Psychological comorbidities like depression, distress, and anxiety can also worsen pain in affected patients^52^ and vice versa.^53^ Associate Professor Strasser differentiated pain in nociceptive pain, including superficial, deep somatic or visceral pain, and neuropathic pain.^54^ Inflammatory mediators, for example, tumour necrosis factor–alpha, cyclooxygenase 2 enzymes, interleukin 1, and interleukin 6, were illustrated as possible pain modulators,^55^ yet more research is needed to better understand the pathophysiology of pain. A daily assessment of pain in patients is necessary and should include questions about pain characteristic, intensity, risk factors, emotional, and cognitive impairments.^56^, ^57^ Associate Professor Strasser emphasized a multimodal pain management^58^ in cancer with pharmacologic interventions^59^, ^60^ and a behavioural approach.^52^ An early prophylactic management of secondary as well as nutrition‐impact symptoms should also be taken in consideration,^61^ as pharmacological side effects like opioid‐induced constipation and nausea can worsen the nutritional status in the affected patients. In this context, Associate Professor Strasser highlighted that an adequate pain management may improve appetite, physical function, quality of life, and medical adherence in cachectic patients with advanced cancer.^62^, ^63^

## Depression

Professor Joan Reid from Belfast, UK, gave an overview about depression, an underestimated burden in cachectic patients. Depression in chronic illnesses may be undetected in 50–60% of cases,^64^ and among the elderly, the incidence might even be higher.^65^, ^66^ In this context, depression is a common comorbidity in CHF patients with a prevalence of 20%.^67^ It is a risk factor for overall poor quality of life,^68^ higher rate of readmissions,^69^ and increased mortality in cardiac patients.^70^ Professor Reid demonstrated that cachexia challenges cancer patients and family members on many different levels: psychologically, socially, and emotionally.^71^ The aggravating psychosocial impact of cancer cachexia on the entire family was observed in a cross‐sectional survey with 702 family members of cachectic patients with advanced cancer—60% of family members reported some kind of eating‐related stress.^72^ In another cross‐sectional study, 306 advanced cancer patients were examined for symptoms that are prone for depression. In 52 patients with severe cachexia due to cancer, sleeping was disturbed in 73% of patients, fatigue was present in 77%, distress in 62%, lack of appetite in 69%, and lack of energy in 62%.^73^ The symptom burden increased with the stage of cachexia. Therefore, Professor Reid urged to raise awareness and understanding for depression in cachectic patients especially among health care professionals.^74^ She appealed for validated identification criteria and management strategies in order to improve the quality of life in these patients. Currently, there are no standardized criteria for diagnosis of depression in cachectic patients. Hence, she promoted two questionnaires to assess mental health: the emotional well‐being subscale from Functional Assessment of Chronic Illness Therapy and the Kidney Disease Quality of Life 36‐Item Short Form Survey. Both of them were used in a longitudinal study on cachexia among patients with renal failure.^75^ Therapeutic approaches of depression in cachectic cancer patients involve serotonin reuptake inhibitors^76^, ^77^ and symptomatic treatment of cachexia in the affected patients. Cytokines were also described as a possible link between depression, cachexia, and chronic diseases and may be further treatment targets.^78^

## Muscle weakness and fatigue

The role and objective assessment of skeletal muscle in cancer cachexia was presented by Assistant Professor Richard Dunne from Rochester, USA. Primarily, skeletal muscle wasting is an important contributor of cancer cachexia leading to decreased physical performance and disability in these patients.^10^, ^79^ In CHF patients, sarcopenia is present in about 20–50% of cases.^80^ Muscle loss with or without weight loss has a worsening impact on functional capacity and quality of life in CHF patients.^81^ Muscle weakness is caused by alterations of muscle morphology and metabolism.^8^, ^82^ Various objective measurements to assess muscle strength and physical performance were discussed: isokinetic dynamometers of the lower limb,^83^ handgrip dynamometry,^84^ stair climbing power test, 6‐minute walk test,^85^ and short physical performance battery test.^86^ Additionally, Professor Dunne pointed out the heterogeneous definitions of cachexia in recent research studies. Still, the most common criteria in the definitions was weight loss.^87^ Low muscle strength, a main component of sarcopenia,^88^ is less frequently included in clinical practice. He concluded that there is no gold standard for assessing strength in cachectic patients,^89^ and therefore, the aforementioned examinations are rarely carried out in clinical practice. Currently, common treatments of muscle weakness include aerobic or resistance exercise^90^ and nutritional support according to the European Society for Clinical Nutrition and Metabolism guidelines.^91^ Several clinical trials with multimodal interventions combined with a nutrition and exercise program were highlighted.^92^, ^93^, ^94^, ^95^, ^96^ The results of these trials showed superiority for multimodal interventions in feasibility, safety, and improvement of physical performance in cachectic patients with advanced cancer.

## Shortness of breath

In the last lecture of the session, Dr Markus Anker from Berlin, Germany, presented shortness of breath as one of main symptoms in cachectic patients. Dyspnoea is frequently observed in patients with heart failure (depending on the stage in up to 100% of patients)^97^ and malignancies (~50%).^98^ In an observational study, even treatment‐naïve colorectal cancer patients showed heart failure‐like symptoms with mildly reduced left‐ventricular ejection fraction and low breath efficiency.^99^ Cancer patients in palliative care frequently suffer from respiratory symptoms including coughing (20–90%), haemoptysis (5–40%), and shortness of breath (30–90%).^100^ Dyspnoea in end‐stage cancer can be aggravated by lung metastases, pleural effusions, or respiratory muscle fatigue due to cachexia.^101^, ^102^ In patients with advanced CHF, shortness of breath (50–100%) and tiredness (60–90%) are very common.^103^ As a possible pathophysiologic explanation for shortness of breath, Dr Anker discussed the ‘muscle hypothesis’ leading to a catabolic metabolism.^104^ This can result in an increased metabo‐ergoreflex^105^ and a sympathetic activation,^106^ possibly explaining higher resting heart rates of patients with mainly advanced stage colorectal, pancreatic, and non‐small cell lung cancer, compared with health controls.^107^ Common diagnostic tests for cachectic patients with dyspnoea include imaging (chest X‐ray and CT), physical examinations (oxygen saturation, blood pressure, and haemoglobin), and functional testing (pulmonary, ergometry, and performance tests).^100^ Dr Anker concluded that regular assessments of dyspnoea including physical, emotional, and social components are important in patients with chronic diseases in order to determine the best therapy for the patient with respect to the underlying disease, including pharmacological and nonpharmacological treatments.^108^

## Conclusions

During the ‘12^th^ International Conference on Cachexia, Sarcopenia and Muscle Wasting’, five lectures were dedicated to the most common clinical problems of cachectic patients with chronic diseases, including swallowing problems, pain, depression, muscle weakness, and shortness of breath. Future consensus definitions regarding these clinical problems will help to identify patients with such problems more efficiently. Only then goal‐oriented therapies can be implemented. Major components for the treatment of these clinical problems include nutritional support, exercise training, talk therapy, and pharmacological treatments.

## Conflict of interest

M.S.A. has received personal fees from Servier, outside the submitted work. All other authors declare no conflict of interest.
